# Navigation accuracy after automatic- and hybrid-surface registration in sinus and skull base surgery

**DOI:** 10.1371/journal.pone.0180975

**Published:** 2017-07-10

**Authors:** Tanja Daniela Grauvogel, Paul Engelskirchen, Wiebke Semper-Hogg, Juergen Grauvogel, Roland Laszig

**Affiliations:** 1 Department of Otorhinolaryngology–Head and Neck Surgery, Medical Center–University of Freiburg, Faculty of Medicine, University of Freiburg, Freiburg im Breisgau, Germany; 2 Department of Oral and Maxillofacial Surgery, Medical Center–University of Freiburg, Faculty of Medicine, University of Freiburg, Freiburg im Breisgau, Germany; 3 Department of Neurosurgery, Medical Center–University of Freiburg, Faculty of Medicine, University of Freiburg, Freiburg im Breisgau, Germany; Universita degli Studi di Palermo, ITALY

## Abstract

**Objective:**

Computer-aided-surgery in ENT surgery is mainly used for sinus surgery but navigation accuracy still reaches its limits for skull base procedures. Knowledge of navigation accuracy in distinct anatomical regions is therefore mandatory. This study examined whether navigation accuracy can be improved in specific anatomical localizations by using hybrid registration technique.

**Study design:**

Experimental phantom study.

**Setting:**

Operating room.

**Subjects and methods:**

The gold standard of screw registration was compared with automatic LED-mask-registration alone, and in combination with additional surface matching. 3D-printer-based skull models with individual fabricated silicone skin were used for the experiments. Overall navigation accuracy considering 26 target fiducials distributed over each skull was measured as well as the accuracy on selected anatomic localizations.

**Results:**

Overall navigation accuracy was <1.0 mm in all cases, showing the significantly lowest values after screw registration (0.66 ± 0.08 mm), followed by hybrid registration (0.83± 0.08 mm), and sole mask registration (0.92 ± 0.13 mm).On selected anatomic localizations screw registration was significantly superior on the sphenoid sinus and on the internal auditory canal. However, mask registration showed significantly better accuracy results on the midface. Navigation accuracy on skull base localizations could be significantly improved by the combination of mask registration and additional surface matching.

**Conclusion:**

Overall navigation accuracy gives no sufficient information regarding navigation accuracy in a distinct anatomic area. The non-invasive LED-mask-registration proved to be an alternative in clinical routine showing best accuracy results on the midface. For challenging skull base procedures a hybrid registration technique is recommendable which improves navigation accuracy significantly in this operating field. Invasive registration procedures are reserved for selected challenging skull base operations where the required high precision warrants the invasiveness.

## Introduction

Computer-aided-surgery (CAS) has rapidly evolved over the last years. Today there is a variety of referenciation and registration modalities as well as several possibilities for instrument navigation. CAS in the field of ENT is established particularly in sinus surgery and is considered a helpful tool by most surgeons [1]. In clinical applications navigation accuracies between 0.5 and 2.77 mm are reported [2–4].

In skull base surgery the use of navigation systems using non-invasive tracking and registration tools is still challenging due to the complex anatomy of the skull base requiring navigation accuracies < 1 mm [5]. Therefore, navigation accuracy studies which allow a clinical transfer of the results are mandatory.

In clinical routine navigation accuracy is mostly tested intraoperatively by pointing at anatomic landmarks or by relying on the predicted accuracy by the navigation system itself (RMSE, root-mean-square error) [6–8]. The variety of existing accuracy parameters is confusing; therefore, a direct comparison of accuracy studies is often not possible. However, in microsurgical procedures a trustworthy measure of accuracy is mandatory. The most clinically relevant error description is the TRE (target registration error) which expresses the distance between the true position of a surgical target and its measured position after registration has been performed [9, 10].

As navigation accuracy is mainly influenced by the registration process [1, 11, 12], the present study compared the gold standard of screw registration with non-invasive image-to-world registration techniques, with a special focus on hybrid registration for the possible improvement of navigation accuracy. Accuracy measurements took place in an experimental setting closely resembling clinical conditions. TRE was measured to express overall navigation accuracy as well as navigation accuracy on defined anatomic localizations which are of special interest for ENT surgeons.

## Material and methods

**Fig 1:**
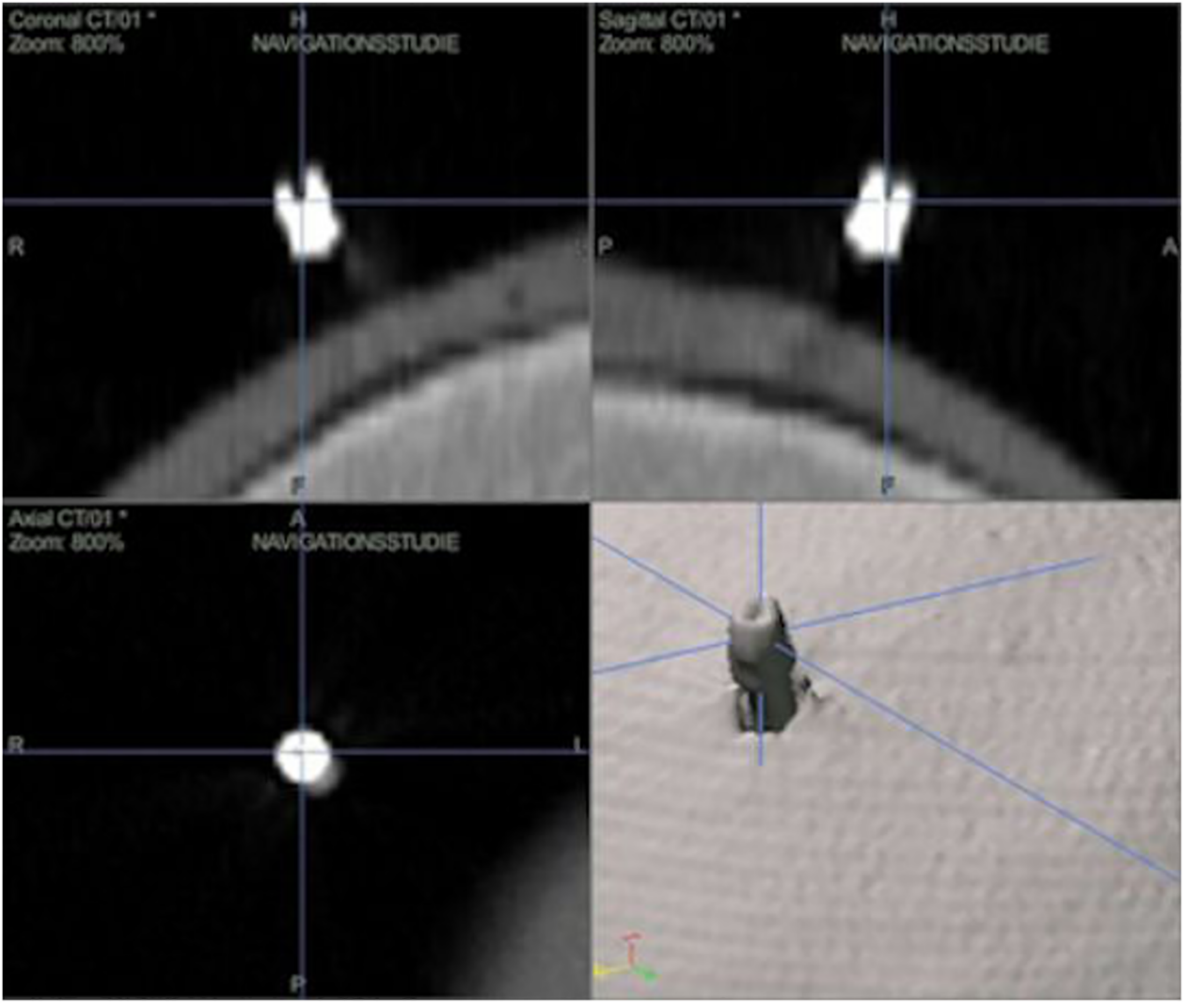
Definition of virtual target points (x_(v)_-, y_(v)_- and z_(v)_-coordinates) in the image data set before skull registration is performed.

For all measurements, an active optical navigation system (Stryker® Navigation System II-Cart, Stryker® Instruments, Kalamazoo, Michigan, USA) was used. Four individually phantom skulls were formed by a 3D printer (Spectrum Z510; Z Corp., Burlington, MA) based on real patient data using a special blend of powder coating, binder and hardening powder. A specially customized silicone skin was formed for skin and soft tissue simulation, taking care that there was no connection to the real patient. Important extra- and intracranial localizations were marked with 26 symmetrically placed invasive titanium screws (Cranial Marker Set, length 5/17 mm, diameter 1 mm, cavity 1 mm, Stryker®-Leibinger, Freiburg, Germany), serving as target fiducials. For internal marker positioning and actuation, a part of the calvarium was left open. CT images (Somatom Sensation 16, Siemens AG, Munich, Germany) of every phantom skull with a layer thickness of 1 mm, gantry 0°, resolution 512 x 512, and a pixel size of 0.396 x 0.396 mm were performed. The virtual _(v)_ target points were then defined in the CT with x_(v)_-, y_(v)_-, and z_(v)_-coordinates (Fig 1).

In the further course of the experiments these points remained unchanged and were used as reference points for all measured values. During experiments the actuation of the target fiducials was performed using a pointer equipped with three LED lamps, resulting in real _(r)_ x_(r)_-, y_(r)_, and z_(r)_- coordinates. The deviation between the measured values and the predetermined reference points (TRE) was determined using the Euclidean distance calculation d_VR_ = $\sqrt{{\left(X_{V}-X_{R}\right)}^{2}+{\left(Y_{V}-Y_{R}\right)}^{2}+{\left(Z_{V}-Z_{R}\right)}^{2}}$.

**Fig 2:**
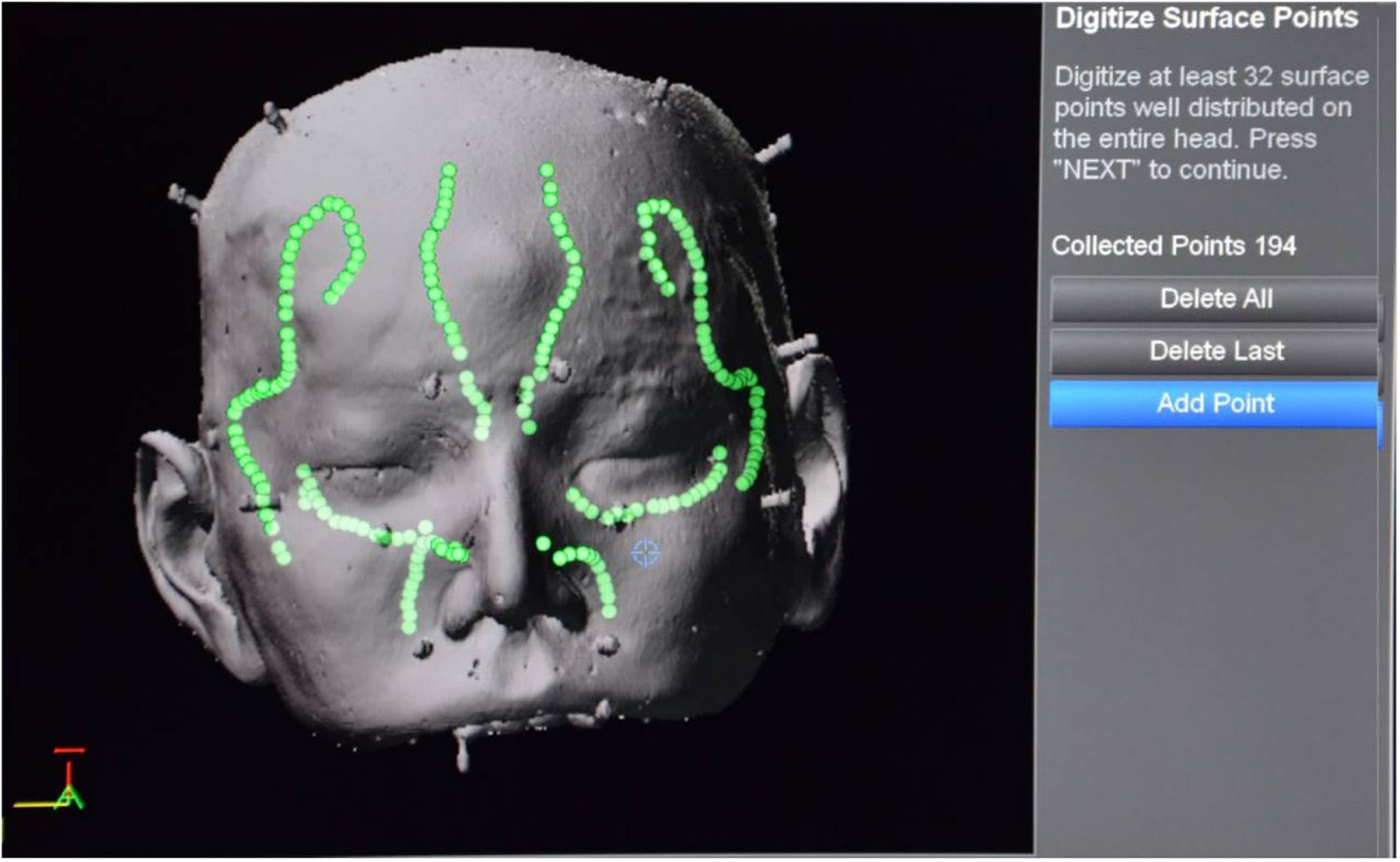
Screenshot after LED-mask registration and additional surface matching (M–M+SM); distribution of points collected with the pointer tip.

Tracking was performed by using a self-adhesive, elastic LED mask (M) in all cases. Altogether, three registration modalities were examined: (1) invasive marker registration (M-IM) as the goldstandard registration serving as a reference with best possible accuracy results, (2) LED mask (M-M), and (3) LED mask plus surface matching (M-M+SM, hybrid registration). For invasive marker registration, four titanium screws were placed in retroauricular and frontoparietal positions bilaterally. While only 10 LEDs on the forehead are necessary for tracking, all 31 LEDs are required for mask registration. In the case of hybrid registration (3) LED-mask registration was complemented by an additional, semi-automatic surface matching. In this process, prominent structures of the surface of the face (nose, cheekbones, orbits and forehead) were–while partially being guided by the configuration of the LED mask–followed with the pointer tip while continuously recording surface points (Fig 2). These additional 200 to 250 surface points were included in further calculations by the navigation system.

All measurements took place in the operating room. Overall navigation accuracy with respect to all 26 titanium screws was calculated. Then four localization groups were allocated (Table 1) and TRE values on single anatomic localizations were measured.

**Table 1:** Overview of screw localization of all groups.

Localization group	Screw localization
**Frontal/parietal**	frontal sinus right and left
	temporal anterior right and left
	temporal posterior right and left
**Midface**	maxillary sinus right and left
	infranasal right and left
	zygomatic arch right and left
**Periauricular**	preauricular right and left
	retroauricular right and left
	mastoid right and left
**Skull base**	ethmoid sinus right and left
	spenoid sinus right and left
	internal auditory canal right and left
	clivus right and left

There were four experimental series (four skull models) per registration group. Each experimental series consisted of five experimental runs. For each experimental run, five values were collected at all target fiducials resulting in 100 measured values for each target fiducial. Altogether, the sample size was n = 100 x 26 x 3 = 7800 values recorded for the three registration methods.

For statistical analysis SAS® 9.2 (SAS® Institute, Cary, North Carolina, USA) was used. One-way ANOVA, repeated-measures ANOVA and Tukey test were performed. Significance was defined as p < .05.

The study was approved by the institutional review board of the medical faculty, Albert-Ludwigs-University of Freiburg, Germany.

## Results

Overall navigation accuracy of 0.66 ± 0.08 mm using invasive marker registration was significantly superior compared to LED-mask registration (0.92 ± 0.13 mm) and LED-mask registration with additional surface matching (0.83 ± 0.08 mm). No significant differences were found between the two non-invasive registration methods.

**Fig 3:**
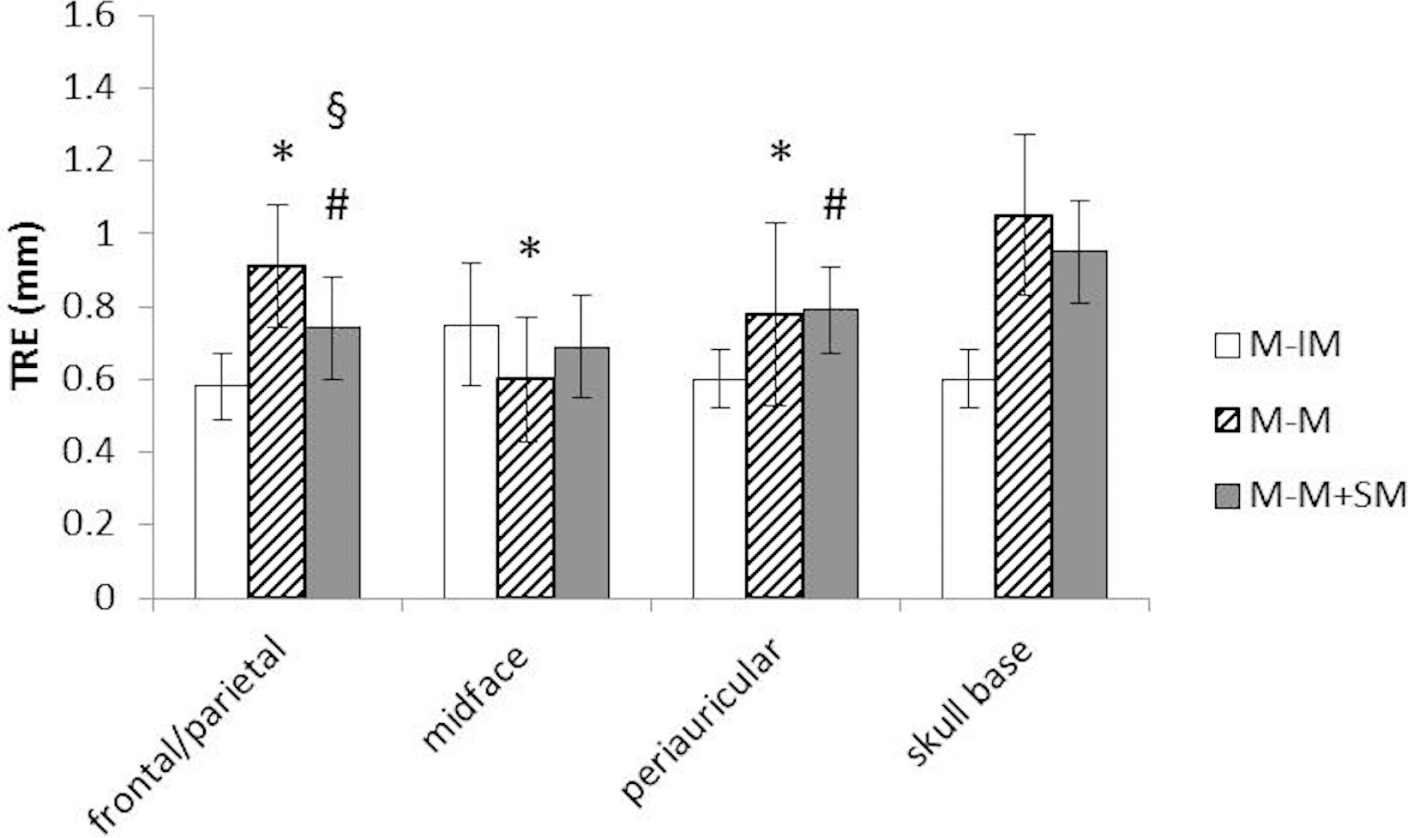
TRE as quadratic mean ± SD in mm; * M-IM versus M-M; ^#^ M-IM versus M-M+SM; ^§^ M-M versus M-M+SM; p < .05, ANOVA, Tukey test.

In terms of the different localization groups, the invasive marker registration showed a significantly higher precision frontal/parietal (0.58 ± 0.09 mm) and periauricular (0.60 ± 0.08 mm) compared to the non-invasive registration procedures (M-M 0.91 ± 0.17 mm, M-M+SM 0.74 ± 0.14 mm frontal/parietal; M-M 0.78 ± 0.25 mm, M-M+SM 0.79 ± 0.12 mm periauricular) (Fig 3). On the midface, single LED-mask registration proved to be more accurate than the gold standard (0.60 ± 0.17 mm vs. 0.75 ± 0.17 mm) (Fig 3). Significant differences between the two non-invasive registration methods could only be registered in the frontal/parietal region (0.91 ± 0.17 mm M-M versus 0.74 ± 0.14 mm M-M+SM) (Fig 3).

**Fig 4:**
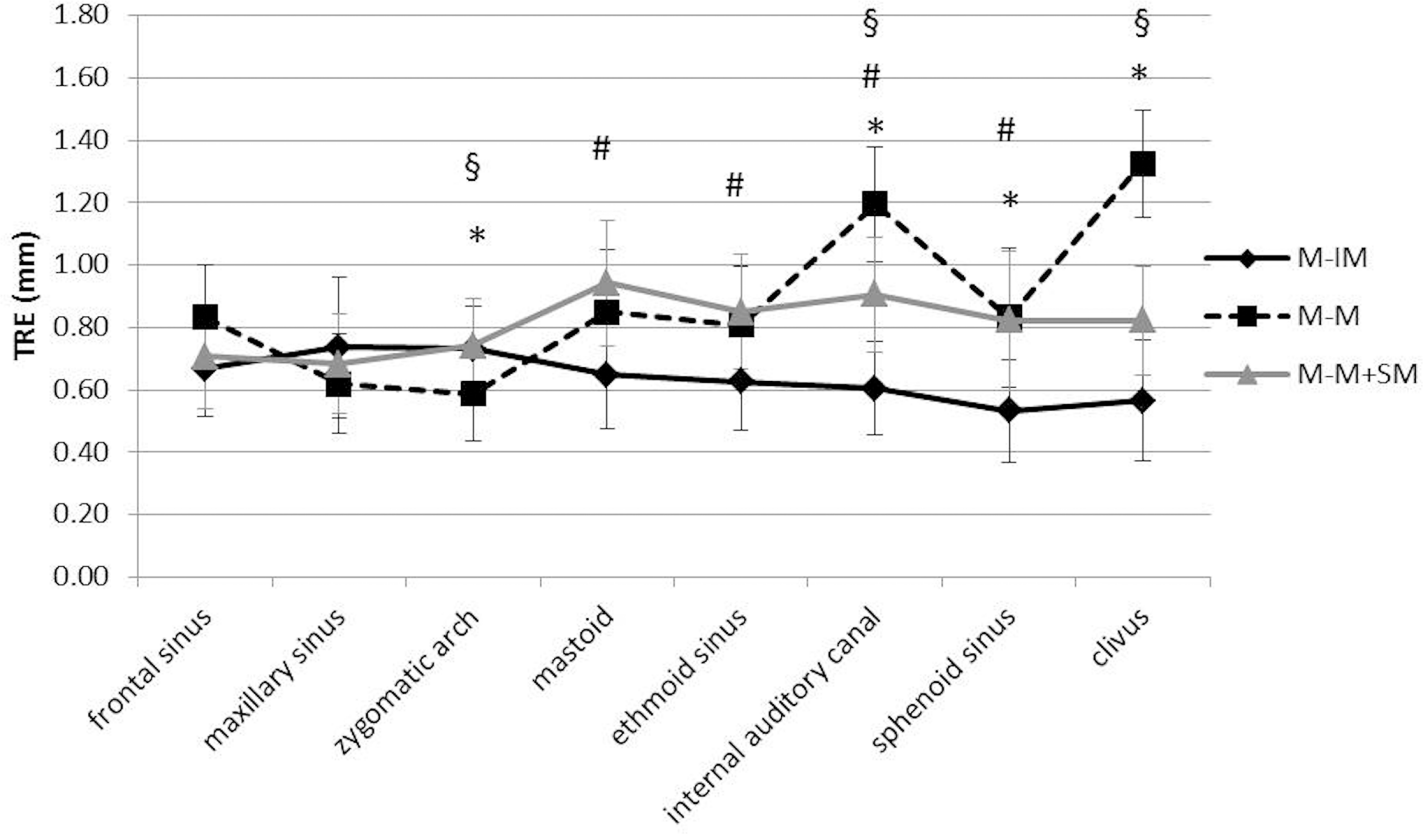
TRE as quadratic mean ± SD in mm; * M-IM versus M-M; ^#^ M-IM versus M-M+SM; ^§^ M-M versus M-M+SM; p < .05, ANOVA, Tukey test.

In terms of the single anatomic localizations invasive marker registration was significantly more accurate than the two non-invasive registration methods on the sphenoid sinus (0.53 ± 0.16 mm IM vs. 0.83 ± 0.24 mm M-M and 0.82 ± 0.22 mm M-M+SM) and on the internal auditory canal (0.60 ± 0.15 mm IM vs. 1.19 ± 0.26 mm M-M and 0.91 ± 0.18 mm M-M+SM) (Fig 4). IM registration was significantly superior on the clivus compared to M-M registration (0.57 ± 0.19 mm vs 1.32 ± 0.39 mm). On the ethmoid sinus (0.63 ± 0.16 mm vs. 0.85 ± 0.18 mm) and on the mastoid (0.65 ± 0.18 mm vs. 0.94 ± 0.20 mm), IM registration was significantly superior compared to M-M+SM registration (Fig 4).

Using LED-mask registration alone, the navigation accuracy was highest on the midface. The smallest deviation of 0.59 ± 0.20 mm could be measured on the zygomatic arch, which was significantly lower than IM registration (0.73 ± 0.14 mm) as well as LED-mask registration with additional surface matching (0.74 ± 0.15 mm) (Fig 4). Values with a similarly high precision were collected on the maxillary sinus (0.62 ± 0.19 mm). However, with increasing distance from the midface, navigation accuracy decreased using LED-mask registration alone. The highest TRE value was measured on the clivus (1.32 ± 0.39 mm).

Combining LED-mask registration with an additional surface matching, navigation accuracy on more distant targets from the midface increased in most cases. On the clivus (0.82 ± 0.17 mm vs. 1.32 ± 0.39 mm) as well as on the internal auditory canal (0.91 ± 0.18 mm vs. 1.19 ± 0.26 mm), precision was significantly higher using additional surface matching compared to single LED-mask registration (Fig 4).

On the frontal and maxillary sinus, all registration modalities showed comparable results without any significant differences.

## Discussion

Refinements in microsurgical techniques have led to an expansion of minimally invasive procedures in head and neck surgery. Simultaneously, the complexity and proximity of critical anatomic structures, particularly on the skull base, requested the intraoperative use of navigation techniques and a constant development of CAS. Thus, the variety of navigation systems and registration technologies is tremendous. Nevertheless, the possibilities of available navigation tools are often not exhausted in clinical routine.

It is known that one of the most important steps in CAS is the registration process, which aligns the image data with the patient´s geometry. This registration process has strong influence on navigation accuracy [13, 14].

The present study examined existing registration modalities of the Stryker® Navigation System II-Cart, a widely used active optical navigation system, with respect to practicability and navigation accuracy. The objective was to find the appropriate non-invasive registration method offering best accuracy results in dependence on the operating field. To achieve best possible clinical comparability, skull phantoms with realistic heterogeneous anatomic geometry were used. Due to the individually customized silicone mask for skin simulation, the skulls had a realistic facial outline with nose and cheek, which is of special interest for surface registration. The opening in the calvarium allowed exact pointer actuation on skull base localizations. In spite of soft tissue simulation due to the individual silicone masks, we are aware that skin surface alterations caused by edema, tumor, or skin turgor could not be imitated by this method.

For skull referenciation a self-adhesive LED mask was used, which can be applied easily and quickly and furthermore allows unimpeded head movement. Another advantage of this LED mask is the possibility to perform an automatic-surface registration procedure. But as the mask has to be affixed on the midface during the whole surgical procedure, its use is subject to restrictions in certain surgeries. For example, referenciation with the self-adhesive LED mask is hardly to achieve in open tumor surgery of the midface or in reconstructive surgery of the midface or frontal skull base in the treatment of extensive midfacial fractures.

The titanium screws serving as target fiducials or in case of invasive marker registration as registration fiducials had a squared hole guaranteeing the best possible identification on the image data sets as well as on the phantoms. However, under clinical conditions, screws have to be implanted before imaging is performed, which is accompanied by discomfort for the patient and, in some cases, additional radiation exposure due to a second scan.

Invasive marker registration was predominantly used as a reference providing best accuracy results. In clinical routine the application of invasive markers is reserved for few navigated surgeries that require a high degree of accuracy that warrants the invasiveness (e.g. cochlear implantation of the malformed inner ear, maxillofacial reconstructions, or co-registration during surgery) [15–17].

Marker localizations retroauricular and behind the hairline were chosen in a way that were cosmetically tolerable while following the guidelines by West and coworkers regarding the fiducial number and arrangement [18].

We measured a high overall navigation accuracy of 0.66 +/- 0.08 mm after screw registration which was significantly superior in comparison to the non-invasive registration procedures.

Pillai et al. reported comparably low TRE values after registration on three titanium screws around a retrosigmoidal craniotomy using a Stryker® Navigation System [19].

The comparison between screw registration (1.96 mm), scalp fiducial registration (3.18 mm) and LED mask registration (3.20 mm) in a cadaver study also showed a statistically greater accuracy when reaching external targets after screw registration. But overall TRE values in that study were higher in comparison to our results which might be explained by differences in study design, particularly by the low number of only ten target fiducials [20]. Furthermore several studies using other navigation systems confirm screw registration still being the gold standard [13, 21–24].

In terms of localization groups and single anatomic localizations, the experimental results after screw registration demand a differentiated interpretation. On the frontal/parietal as well as on the periauricular regions, invasive marker registration was significantly superior to the non-invasive registration modalities, probably due to the proximity of registration and target fiducials. Additionally, on skull base targets, IM registration generated the best possible accuracy results, which might be due to its favorable geometrical fiducial arrangement. In contrast to these results, values on the frontal and on the maxillary sinus showed no significant differences between all registration modalities.

LED mask registration showed an overall navigation accuracy of 0.92 mm. Other studies report accuracies between 2.16 mm and 3.2 mm depending on the localization and the number of target fiducials [20, 21].

In terms of single anatomic localizations, LED mask registration showed the best accuracy on the midface. This high navigation accuracy is used in cranio-maxillofacial procedures, e.g. reconstruction of orbital fractures with titanium meshes [25].

More distant targets from the midface, showed worse TRE values but they could be improved by additional surface matching (frontal, sphenoid sinus, clivus).

Differing results revealed the work of Makiese and coworkers measuring higher navigation accuracy on internal targets than on external targets when using LED mask registration [20]. A probable explanation for these contrary results is that their locations of external target fiducials (frontal, parietal, and occipital) were further afar from the midface location of the registration mask. Moreover, they used only ten target fiducials for accuracy evaluation.

Data referring to navigation accuracy on single anatomic localizations when using LED mask registration are sparse. Makiese et al. measured a target registration error of 2.4 mm on the clivus after LED mask registration (1.32 mm in the present study) [20]. A neurosurgical study reported a 98% success rate of a biopsy needle passing through a 4 mm circular target in the middle cranial fossa [26].

There is limited data in the literature for hybrid registration techniques. In the present study the combination of LED mask registration and pointer-based surface matching led to higher navigation accuracy on the clivus (0.82 ± 0.17 mm vs. 1.32 ± 0.39 mm) and on the internal auditory canal (0.91 ± 0.18 mm vs. 1.19 ± 0.26 mm) compared to LED mask registration alone. Greenfield and coworkers used laser surface scanning with additional landmark registration in endonasal transsphenoidal surgery and reported a high application accuracy [27]. On the lateral skull base the combination of pair-point matching and surface matching improved navigation accuracy in a cadaver study (1.58 mm vs. 1.49 mm) [5]. Another study group could increase navigation accuracy in this area by combining fiducial and landmark registration [28].

## Conclusion

Overall navigation accuracies below 1 mm could be reached in the experimental setting with invasive and non-invasive registration modalities. LED mask-based referenciation and registration can be considered a suitable alternative in clinical application. Navigation accuracy on different anatomic regions may vary considerably and is not easily predictable. There is a strong correlation between site-specific navigation accuracy, the selected registration modality, and the number and distribution of registration fiducials in three-dimensional space. LED mask registration showed the best accuracy results on the midface. The challenge to achieve sufficient navigation accuracy with non-invasive registration modalities on the lateral skull base can be better addressed by using hybrid registration techniques. Invasive registration techniques remain a back-up tool for challenging skull base procedures requiring best possible navigation accuracy results.

## Supporting information

S1 Supporting Information Dataset. (XLSX)
